# Participation in eHealth Communication Interventions Among Patients Undergoing Hemodialysis: Scoping Review

**DOI:** 10.2196/51900

**Published:** 2024-11-11

**Authors:** Anne Deinboll, Cathrine Fredriksen Moe, Mette Spliid Ludvigsen

**Affiliations:** 1 Faculty of Nursing and Health Sciences Nord University Mo i Rana Norway; 2 Faculty of Nursing and Health Sciences Nord University Bodø Norway; 3 Department of Clinical Medicine Randers Regional Hospital Aarhus University Aarhus Denmark

**Keywords:** eHealth, electronic health records, hemodialysis, patient participation, renal dialysis, renal insufficiency, chronic, mobile phone

## Abstract

**Background:**

eHealth communication interventions have been shown to offer individuals with chronic kidney disease the opportunity to embrace dialysis therapies with greater confidence, the potential to obtain better clinical outcomes, and an increased quality of life. eHealth is an emerging field that offers diverse, flexible designs and delivery options. However, existing evidence on eHealth communication among patients undergoing hemodialysis is sparse and scattered and lacks systematization.

**Objective:**

This scoping review aims to identify and map the current evidence on patient participation in eHealth communication interventions. We aimed to map the associations between interventions and electronic health records, the participative role of individuals living with chronic kidney disease and undergoing hemodialysis, and the barriers to and facilitators of patient involvement in eHealth communication with health care professionals.

**Methods:**

This study used the Joanna Briggs Institute methodology for conducting a scoping review. Studies eligible for inclusion were those that included adult patients (aged >18 y) undergoing all types of hemodialysis, including prescheduled in-center hemodialysis and conventional home-based hemodialysis. Systematic searches were completed in Ovid MEDLINE, Ovid Embase, EBSCOhost CINAHL with Full Text, Scopus, and ProQuest Dissertations and Theses. Extracted data from the included studies were presented in figures and tables along with descriptions that responded to the research questions. This review was reported according to the PRISMA-ScR (Preferred Reporting Items for Systematic Reviews and Meta-Analyses extension for Scoping Reviews) guidelines.

**Results:**

In total, 9 peer-reviewed studies were included. The main result was a low participative patient role and a vaguely described link to electronic health records. The key participative facilitators were availability of and access to the intervention; security, trust, and confidence; patient knowledge of their health situation and use of self-care; and patient preparedness for an uncertain future health situation and the ability to relate to family and friends about it. The key participative barriers were lack of availability of and access to information, mistrust and lack of safety, lack of knowledge of health situation and self-care, and relational issues. All barriers and facilitators were related to health literacy.

**Conclusions:**

This scoping review summarizes 4 specific and 3 nonspecific eHealth communication interventions developed and evaluated in various studies involving patients receiving hemodialysis. A knowledge gap exists between low levels of patient participation in eHealth communication and patients’ limited access to electronic health records. eHealth communication interventions should implement patient participation and focus on the fact that different modalities of eHealth communication can complement face-to-face communication.

**International Registered Report Identifier (IRRID):**

RR2-10.2196/38615

## Introduction

### Background

Despite substantial improvements in hemodialysis delivery and outcomes over the last decade, patients undergoing hemodialysis continue to have negative experiences [1]. Particularly, patients undergoing hemodialysis still experience poor quality of life (QOL); their symptoms remain prevalent; and their financial burden, morbidity, and mortality remain unacceptably high [2]. Studies have indicated a potential survival advantage with intensive dialysis (ie, an increase in dialysis frequency or duration), a goal that can be achieved with home-based dialysis [3]. Home hemodialysis (HHD) is mostly provided in Western Europe, and increased use of HHD aims to strengthen patients’ position and QOL and reduce financial burdens and human resource challenges [2,4]. Patients undergoing hemodialysis depend on effective and real-time communication with renal health care professionals (HCPs) to optimize their QOL owing to the high symptom burden and the complexity of the illness and treatment [5].

The increasing demand for participation in health-related decision-making in both in-center hemodialysis and HHD requires innovative interventions for eHealth communication [6]. Digital home monitoring is in development and enables 2-way communication between patients and HCPs. Many technical challenges have been described in implementing communication interventions and information flows in digital health care systems [7-9]. However, the challenges of the participative role of patients undergoing hemodialysis have rarely been explored.

Approximately 3.9 million individuals living with stage-V chronic kidney disease (CKD) worldwide receive kidney replacement therapy, kidney transplantation, or various forms of dialysis, and the numbers continue to rise [10,11]. In-center hemodialysis is more common than peritoneal dialysis (PD), with approximately 2.3 million individuals undergoing hemodialysis in 2019 [12]. In hemodialysis, blood is pumped out of the body to an artificial kidney machine and returned to the body via tubes connecting the machine to the patient [13], thus involving direct access to the patient’s circulatory system. Complications such as severe bleeding, venous thrombosis, infection, or low blood pressure may occur; therefore, patients should be knowledgeable and competent regarding interventions to reduce complications and ensure their safety [14]. Chuasuwan et al [15] reported a lower QOL in patients receiving hemodialysis than in those receiving PD. PD can be performed more easily than hemodialysis at home [15].

Patient participation is increasingly promoted to improve HCPs’ responsiveness to patient needs and ensure the legitimacy of decisions affecting patient care [16,17]. Patient participation has individual and collective dimensions. The individual dimension refers to enabling patients to have more influence over their health by increasing their capacity to gain more control over the issues they consider important, including access to their electronic health record (EHR). The collective dimension refers to patient participation in collective activities, in which patients, relatives, representatives, or service users are actively engaged in shaping the development of health care services [18]. The expanding development and dissemination of eHealth interventions is a paradigm shift toward enhanced individuality and patient-centered care [15-17].

Interactions between nurses and patients during hemodialysis sessions involve ongoing dialogues on topics such as hydration status, treatment goals, and practical decisions regarding dialysis procedures [19]. These interactions differ significantly from those in other medical contexts, such as diabetes, owing to frequent and prolonged treatment durations. A critical aspect for patients undergoing hemodialysis involves ongoing consultations regarding health status and eligibility for kidney transplantation, a life-saving procedure for recipients. Inadequate communication about transplantation status between patients and the involved clinicians can compromise patient empowerment and the nurse-patient relationship [20]. Moreover, a proportion of patients receiving hemodialysis do not qualify for kidney transplantation owing to various medical factors [2]. Considerations such as dialysis discontinuation, end-of-life decisions, and subsequent adjustments to supportive treatment and care preferences become particularly crucial [19]. This highlights another significant issue that profoundly impacts this patient group. We hypothesized that individuals undergoing hemodialysis encounter unique issues and interactions owing to the long-term collaboration and communication between patients and HCPs during frequent dialysis sessions whether in a clinical setting or at home. Overall, these factors may necessitate specific eHealth communication interventions tailored to the needs of patients receiving hemodialysis. Therefore, we found it appropriate to focus exclusively on the population undergoing hemodialysis.

### Prior Work and Implications for This Study

A previous review reported a low level of eHealth literacy when assessing the availability, acceptability, and use of mobile health in a population with CKD [21]. Another review found a knowledge gap in sociocultural and safety aspects when exploring mobile health use in a population undergoing hemodialysis [22]. However, none of these reviews addressed patient participation, communication, barriers, and facilitators. Therefore, this study explored the participative role of patients undergoing hemodialysis. We used the phrase *eHealth communication* to refer to communication technologies that enable HCP-patient interaction through electronic means [23]. This term encompasses digital and electronic communication interventions [8]. Many eHealth interventions are multimodal, and their definitions may overlap [6]. Distinguishing between *synchronous communication* as video, telephone, and direct messaging functionalities and *asynchronous communication* as email and SMS text messages is common [24]. This study explored the role of patients undergoing hemodialysis in both types of eHealth communication, such as patient portals; telehealth solutions (eg, videoconferencing); and the use of computers, minicomputers, tablets, networks, or cloud storage for managing and storing medical records.

Furthermore, we explored the potential of 2-way digital communication in patient outcomes. Electronic patient-reported outcome measures (ePROMs) improve patient outcomes [25,26]. Knowledge of patient experience with digital communication related to the use of ePROMs is lacking [27]. In addition, we aimed to investigate the role of patients in digital communication related to ePROMs. The COVID-19 pandemic boosted the development and use of eHealth in nephrology; however, additional knowledge of the barriers to and facilitators of patient participation is required owing to the existing knowledge gap regarding why some patients do not use digital communication. Phelps et al [28] analyzed the use of a UK patient portal from 2009 to 2013 by patients with CKD and found that inactive users had not registered with or logged on to the patient portal. A study of barriers to and facilitators of the use of general eHealth by older adults found that high use of eHealth by older people was not possible [29]. Therefore, we found it necessary to explore the need for digital communication in patients undergoing hemodialysis. A comprehensive overview of the available eHealth communication interventions for patients undergoing hemodialysis and HCPs will aid in identifying the status of patient participation in eHealth communication and the gaps and areas for further development.

### Objectives and Research Questions

This scoping review aimed to systemize and map emerging research on eHealth communication interventions for patients undergoing hemodialysis and their participative role in these interventions and to identify barriers to and facilitators of patient participation.

In this review, we were guided by the following research questions (RQs):

1. Which types of eHealth communication interventions for patients undergoing hemodialysis and HCPs can be identified in the literature, and how are they linked to EHRs?
2. Which participative roles of patients undergoing hemodialysis in eHealth communication with HCPs can be identified in the literature?
3. What are the key participative barriers to and facilitators of eHealth communication with HCPs that are encountered by patients undergoing hemodialysis?

This review considered studies that included a population of adult patients (aged >18 y) undergoing hemodialysis. All types of hemodialysis, including prescheduled in-center dialysis and conventional HHD, were included. Studies focusing on patients aged <18 years, patients with stage-I to stage-IV CKD, patients undergoing transplants, and those undergoing PD were excluded. Intervention studies that included patients undergoing hemodialysis and PD were included; however, studies that included only patients undergoing PD were excluded. This review included studies that explored a 3-fold concept. First, the types of eHealth communication interventions included both electronic and digital technology for oral or written communications for example, EHRs (including standardized nursing terminology, electronic patient records or portals, electronic conferencing, and mobile written or oral communication mediated by electronic or digital means). Studies on eHealth interventions with no possibility for communication between patients with CKD and health professionals were excluded (eg, mobile apps for self-efficacy). Second, patient participation in this review referred to the definition by Thompson [30], that is, patient participation requires professionals to engage in 2-way communication. We included studies at all the continuum levels of patient participation, involvement, and similar concepts. Third, barriers to patient participation in eHealth communication included problems, issues, challenges, and obstacles to participation. Facilitators of patient participation included recommendations, interventions or programs, motivation, and experienced results [6]. The barriers and facilitators encountered by patients undergoing hemodialysis were included.

The context for the review was hemodialysis care.

## Methods

### Design

This review was conducted based on an a priori published protocol [31]. We adopted the Joanna Briggs Institute methodology for scoping reviews to present methodological rigor and transparency [32,33]. This scoping review followed a structured and predefined process that requires rigorous methods to ensure that the results are reliable and meaningful to end users, in line with those of other systematic reviews. We conducted a comprehensive systematic literature search, data extraction, and charting accompanied by a narrative summary of eHealth communication interventions and participative roles of patients undergoing hemodialysis. Scoping reviews do not offer direct recommendations for practice; therefore, no critical appraisal of the methodological quality of the included studies was required. However, the results of scoping reviews are widely used to inform the development of trustworthy clinical guidelines [34]. Thus, this review contributes with an overview of existing research on eHealth communication in hemodialysis and future interventions in this field. This review was reported according to the PRISMA-ScR (Preferred Reporting Items for Systematic Reviews and Meta-Analyses extension for Scoping Reviews) guidelines [35]. PRISMA checklist has been provided in Multimedia Appendix 1.

### Eligibility Criteria

The eligibility criteria for this review are outlined in Textbox 1.

**Inclusion and exclusion criteria. Eligibility criteria for the screening and inclusion process, including article type, study type, and language.**

**Inclusion criteria**

- Article and study type: peer-reviewed primary studies, including qualitative, quantitative, and mixed methods study designs, and PhD theses (PhD theses were included due to the suspected empirical and methodological level in line with peer-reviewed articles; Master’s theses, unpublished studies, and conference abstracts were excluded due to lack of peer review)
- Language: English

**Exclusion criteria**

- Article and study type: conference abstracts, unpublished studies, and Master’s theses
- Language: languages other than English

### Information Sources

We performed a systematic search using the following electronic databases: MEDLINE (Ovid), Embase (Ovid), CINAHL (EBSCOhost CINAHL with Full Text), Scopus, and ProQuest Dissertations and Theses Open. No restrictions on year range were imposed on the search owing to the novelty of the research field.

### Search

A full electronic search strategy for MEDLINE (Textbox 2) was developed and adjusted to the remaining databases. The systematic searches were conducted on May 6, 2022. An identical and updated systematic search was conducted across all databases covering the period from January 1, 2022, to June 4, 2024, to ensure the inclusion of the latest research.

**Search documentation for MEDLINE.**

**MEDLINE (Ovid) search strategy**

- Search dates: May 6, 2022 (190 records retrieved); June 4, 2024 (49 new records retrieved; published since May 6, 2022)
- Participant and context search terms were mutually inclusive. Concept search terms were divided into 2 search blocks.
- No limitations
- Searched: (1) population, context, and Medical Subject Heading (MeSH) terms+text words (TW); (2) concept 1, MeSH terms+TW; (3) concept 2, MeSH terms+TW; (4) 1 AND 2 AND 3

We performed backward citation chasing by reviewing the reference lists of the included records and forward citation chasing of the included records using Google Scholar to ensure a complete selection of studies for the review and that no studies were overlooked [36].

### Selection of Sources

All identified records were uploaded to the reference management tool EndNote (version 20; Clarivate Analytics) [37]. Duplicates were removed, and the remaining records were uploaded to the screening software tool Rayyan (Qatar Computing Research Institute) [38]. Initially, the review team conducted a pilot test to ensure consensus on including the first 25 records. Next, titles and abstracts were screened, followed by a full-text screening. We contacted the corresponding authors of 6 studies [39-44] for additional studies or study information to fulfill the study selection process.

The first and second authors discussed the studies during the selection process, and the last author was included in the discussion for agreement on the final included articles.

### Data Charting and Items

A revised charting form from the protocol was used to extract data from the included studies. The data were extracted by the first author, and the extracted data were validated against the eligibility criteria by the second author. The study characteristics and data for each RQ were subsequently extracted.

### Analysis and Presentation of Results

A scoping review intends to provide an overview rather than synthesize data; nevertheless, a basic analytic framework is required to collate, develop, and present a narrative description of the data answering the RQs [32]. The first 3 phases of the basic qualitative coding framework by Elo and Kyngäs [45] were used to consider the level of patient participation according to the taxonomy by Thompson [30]. In addition, this coding framework was used to identify and clarify barriers and facilitators related to participation in eHealth communication interventions available for patients receiving hemodialysis. Disagreements between the first and second authors were resolved based on consensus through discussion or by including the third author. The reviewer team regularly met during several stages of the review process. The extracted and analyzed data from the included papers were presented in figures and tables along with descriptions that responded to the RQs. The tables were presented according to the distribution of sources of evidence by year of publication, country of origin, hemodialysis context, population, aim, and research methods; type of intervention, association with EHR, type of communication, and level of involvement; facilitators; barriers; and a descriptive summary of the charted results.

## Results

### Process of Inclusion of Sources

**Figure 1:**
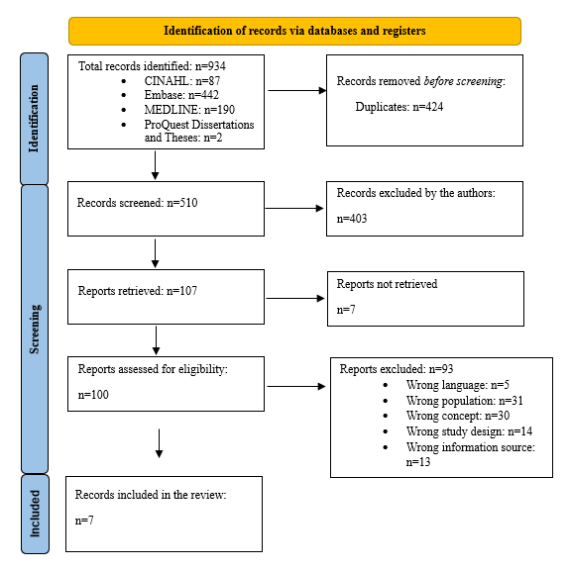
PRISMA (Preferred Reporting Items for Systematic Reviews and Meta-Analyses) flow diagram showing the identification of sources from databases and the screening and inclusion processes [46].

Among the 934 records identified through the systematic search, the titles and abstracts of 510 (54.6%) were screened against the eligibility criteria after removing duplicates (Figure 1 [46]). The full texts of 1.4% (7/510) of the reports [44,47-52] could not be located. In total, 19.6% (100/510) of full-text articles were reviewed. A total of 7 studies on patients’ role in communicative eHealth interventions for patients receiving hemodialysis and HCPs were included. The updated search showed an increase in the number of published studies in this area over the 2 years since the first systematic search in 2022. The search led to the inclusion of 2 additional studies. A total of 9 studies were included.

### Study Characteristics

The 9 studies [41,44,53-59] (Table 1) were published between 2013 and 2023 and were conducted in North America (United States: n=4, 44%; Canada: n=1, 11%), Australia (n=1, 11%), and Europe (United Kingdom: n=3, 33%). The included sources were all peer-reviewed primary studies, and 11% (1/9) of the studies were published as an editorial letter. In total, 56% (5/9) of the studies included participants from only 1 dialysis unit [53-57], whereas 33% (3/9) of the studies included participants from 2 to 4 dialysis units [41,44,58]. A total of 11% (1/9) of the studies were conducted in the transition from a large dialysis unit to HHD [59]. The studies were all conducted in a context involving hemodialysis, varying from small satellite units to larger central hospital units.

**Table 1:** Study characteristics. Information about the authors, year of publication, actual hemodialysis (HD) context and participants, aims, study designs, and methods for the 9 studies.

Study and country	HD context	Participants or population (number, age, and sex)	Aim	Study design and method
Chu et al [59], 2023, Australia	Home dialysis (HD and PD^a^) and carers	34 surveys (HD: 8 patients; PD: 19 patients; 7 carers); 21 interviews with patients (HD: 7; PD: 14; 2 of the interviews were together with carers); aged 49-81 years; both sexes	To explore factors influencing the behaviors and perceptions of patients undergoing home dialysis and their carers regarding telehealth-assisted home visits	A concurrent mixed methods design; a combination of surveys and qualitative, semistructured interviews via telephone
Giles et al [58], 2017, United Kingdom	4 renal units including different modalities of renal treatment (2 central hospital units and 2 satellite units)	Phase 1: 57 participants^b^ of both sexes aged 34-76 years, including 29 patients (other participants: carers and relatives and HCPs^c^); phase 2: 34 participants^b^ aged 18-86 years, including 20 patients (other participants: staff, relatives, and 1 non–English-speaking patient)	(1) To explore the feasibility of patients with renal disease, HCPs, and researchers working in partnership to develop a patient-led quality and safety feedback system within an existing EHR^d^ (RPV^e^); (2) to adapt an existing technology (RPV) to accommodate a patient-led quality and safety feedback system and collect a range of qualitative data from patients and staff to establish the main principles and components of the prototype and assess usability	Qualitative design—an evaluation of the first two phases of an eHealth implementation (1) focus group interviews (separate and combined; patients and staff) and (2) individual interviews
Hazara and Bhandari [53], 2016, United Kingdom	1 renal department	69 patients with CKD^b,f^ and 16 patients receiving HD; aged 20-83 years; both sexes	To elucidate why some users of the self-management and education website RPV remain inactive	Quantitative design; paper-based questionnaires, including a free-text box
Hudson et al [54], 2020, United Kingdom	1 HD unit (on the southeast coast)	10 adult patients (8 undergoing HD and 2 undergoing transplant)^b^; median ages of 54 years for HD and 63 years for nonusers of RPV; both sexes	To understand how patients with kidney disease use PatientView in their self‐care practice	Qualitative design—ethnography; semistructured interviews and observation
Jakubowski et al [55], 2019, United States	A single dialysis clinic	10 adult patients receiving HD (10 initially participated, and 8 patients completed the study); mean age 58.7 years; both sexes	To pilot the feasibility and acceptability of a technology-assisted cognitive behavioral therapy intervention for patients undergoing HD, share design and implementation lessons learned, and provide preliminary results on changes in selected patient-reported symptoms	Mixed methods design—a single-center pilot feasibility study; questionnaire combined with open-ended questions
Kiberd et al [57], 2018, Canada	1 multidisciplinary home dialysis clinic; PD and HD	41 adult patients (HPD^g^ and HHD^h^)^b^ provided consent to join the portal. A total of 27 patients (66%) created a web account and joined the portal. At 6 months, 16 patients had completed follow-up, and at 12 months, 10 patients had completed follow-up for the primary outcome. Mean age 57.1 years; both sexes.		Quantitative design—a single-arm pilot, clinical trial; 4 different questionnaires and in addition the telephone use was compared before and after the patients joined the web portal.
Ladin et al [41], 2021, United States	4 nephrology sites across the United States^i^	Older patients with stage-4 or stage-5 CKD^b^. Of the 60 interviews, 19 (32%) were with clinicians, 30 (50%) were with patients, and 11 (18%) were with care partners; 16 clinicians (84%) were nephrologists; aged ≥70 years; both sexes	To identify the perceptions of patients, care partners, and nephrologists regarding the patient-centeredness, benefits, and drawbacks of telehealth compared with in-person visits	Qualitative design; semistructured interviews
Lew et al [44], 2023, United States	2 HD units in a district on the southeast coast	94 adult patients receiving HD; mean age 56.5 (SD 13.5) y; both sexes	To evaluate patient experiences with the use of telehealth by their nephrologist for HD during the COVID-19 pandemic	Quantitative design; survey using a Likert scale
Minatodani and Berman [56], 2013, United States	1 outpatient dialysis center; HD	33 participants: 30 patients receiving HD and 3 caregivers; aged 37-87 years; both sexes	(1) To evaluate patients’ perceived effectiveness and satisfaction with home telehealth self-monitoring and remote care nurses; (2) to identify perceived facilitators and barriers encountered with remote technology use	Mixed methods design; semistructured individual interviews and an additional survey

^a^PD: peritoneal dialysis.

^b^Mixed population of participants. Patients undergoing hemodialysis were included; however, it was not possible to extract data only from patients receiving hemodialysis.

^c^HCP: health care professional.

^d^EHR: electronic health record.

^e^RPV: Renal Patient View.

^f^CKD: chronic kidney disease.

^g^HPD: home peritoneal dialysis.

^h^HHD: home hemodialysis.

^i^Patients were initially excluded if they were on dialysis, but certain patients initiated hemodialysis during the course of the study and were followed up on until the study ended (a maximum of 18 months).

The studies involved a total of 422 participants, including *others* (eg, HCPs or patients with CKD in varying stages). Overall, 67% (6/9) of the studies included 171 adult patients undergoing hemodialysis [44,53-56,59]; 22% (2/9) of the studies included 79 patients with CKD, including a nonapplicable fraction of patients receiving hemodialysis [41,58]; 11% (1/9) of the studies included 55 patients on dialysis in transition between unit and home dialysis [59]; and 11% (1/9) of the studies included 10 patients on HHD (hemodialysis or PD) [57]. The extractable distribution of patients undergoing hemodialysis was 208 men and 140 women. The number of participants ranged from 8 [55] to 94 [44]. In total, 11% (1/9) of the studies included older patients with CKD aged >70 years [41]. A total of 78% (7/9) of the studies explored the experiences of various populations, including patients with diverse stages of CKD [41,53,54,56-59]. We included these studies because they included patients undergoing hemodialysis, although separating their experiences from those of patients with CKD was not possible. Most of the participants in 33% (3/9) of the studies were undergoing hemodialysis [44,54,56]. Some studies (4/9, 44%) additionally included the experiences of others (caregivers or family) [41,56,58,59] and HCPs [41,58]; nevertheless, the experiences of the patients undergoing hemodialysis were exclusively extracted and separated from the experiences of others. A total of 44% (4/9) of the studies aimed to understand, improve, or provide results on whether patients experienced changes in self-care practices or health-related QOL [41,54-57]. One study elucidated why some users remained inactive [53]. One study aimed to explore factors influencing the behaviors and perceptions of patients on HHD and their carers regarding telehealth-assisted visits and identify the factors influencing their engagement with telehealth [59]. The study was guided by the Behavior Change Wheel framework [60]. One study aimed to evaluate patient experiences with the use of telehealth during the COVID-19 pandemic [44]. One study aimed to evaluate and assess the usability of a patient-led existing technology [58]. The study designs included qualitative (3/9, 33%) [41,54,58], mixed methods (3/9, 33%) [55,56,59], and quantitative (3/9, 33%) [44,53,57] designs. Data collection methods included focus groups and individual oral interviews or questions (6/9, 67%) [41,54-56,58,59], written questionnaires (5/9, 56%) [44,53,56,57,59], and participant observations (1/9, 11%) [54].

### Types of eHealth Communication Interventions, Associations With EHRs, and Level of Patient Participation

#### Overview

A total of 7 eHealth communication interventions were identified. In total, 57% (4/7) were dedicated and named patient portals or eHealth communication platforms: Renal Patient View (RPV), which was investigated in 33% (3/9) of the studies [53,54,58]; RelayHealth [57]; VitelCare Turtle 500 (Vitel Health) [56]; and Vidyo [55]. A total of 43% (3/7) of the interventions were broadly described, unnamed, or general oral video or telehealth consultations [41,44,59] (Table 2).

**Table 2:** eHealth communication interventions. Information about the eHealth communication interventions and their associations with electronic health records (EHRs), possible communication types, and patient participation level [30].

Study	eHealth communication intervention	Association with EHR	Type of communication	Level of patient participation [30]^a^
Chu et al [59], 2023	Telehealth-assisted home visits; telephone or videoconference; telehealth system or platform; —^b^	—	Synchronous	2
Giles et al [58], 2017	An electronic quality and safety feedback system within the web-based patient portal RPV^c^	Link to the EHR for the electronic feedback system; —; yes	Asynchronous	1
Hazara and Bhandari [53], 2016	RPV, a self-management and education website	Yes	Asynchronous	1
Hudson et al [54], 2020	PatientView (formerly termed RPV), a patient portal	Yes	Asynchronous	1
Jakubowski et al [55], 2019	Vidyo videoconferencing platform	—	Synchronous and prearranged	2
Kiberd et al [57], 2018	RelayHealth (McKesson Canada), a web-based eHealth patient portal	Yes	Asynchronous	2
Ladin et al [41], 2021	Telehealth consultations with video and sound	—	Synchronous and prearranged	2
Lew et al [44], 2023	Telehealth consultations, video, and sound via laptop or tablet through a secure video platform	—	Synchronous and spontaneous	2
Minatodani and Berman [56], 2013	VitelCare, a home telehealth monitoring unit with concomitant remote care nurse support	—	Asynchronous, synchronous, and prearranged	2

^a^4=informed decision-making; 3=professional as agent; 2=consultation; 1=information giving; 0=exclusion.

^b^Not applicable.

^c^RPV: Renal Patient View.

#### RPV Patient Portal

This is a patient portal offered for free, initially piloted and implemented in 2004 and intended for use by patients with renal disease under the care of renal physicians in the United Kingdom. RPV is described as a self-management and education website available anywhere with an internet connection using either an internet browser or a dedicated app. Access to the RPV is strictly controlled through usernames and passwords. Patients can authorize access to their accounts by other HCPs involved in their care (eg, general practitioners or community nurses) [53].

The intervention was renamed PatientView in 2018 and has been implemented and used by several other diagnostic groups. RPV is a common UK eHealth portal used in 90% of all renal units and has been widely implemented since 2008 [54]. We were unable to extract specific information on the functionalities of the patient portal. However, information from the RenalView website indicated the following: the portal provides patients with 3 functions—“Manage” (eg, view blood tests with graphs and trends), “Monitor” their health, and “Message” their health care team [61]. The “Manage” and “Monitor” functions are used by patients, whereas the “Message” function was not clearly described [53,54,58].

#### RelayHealth

This is a web-based eHealth patient portal that allows patients and HCPs to communicate through a secure, password-protected application. The portal permits the visualization of the messaging histories of the patient and provider. Specialty nurses are trained in portal use. After entering the portal, the patient and home dialysis health care team, which includes nurses, a home dialysis physician, and dieticians, can send messages related to patient care at any time. Messages can be sent between the patient and health care team, including proposed changes to medication, instructions after a clinic visit, upcoming tests, times of new appointments, and questions about care. Messages are electronically stored within the portal, printed, and placed in the patient’s hospital chart to comply with the hospital’s legal standards. Both the patients and the health care team are made aware of new messages through email prompts [57].

#### VitelCare (Vitel Health)

This is a home telehealth monitoring unit that is compliant with Health Insurance Portability and Accountability Act and is broadband enabled [56]. We were unable to extract information on the monitoring unit from the study. The following information about the intervention is from a previous randomized controlled trial: accessories included a blood pressure monitor, scale, pulse oximeter, glucose monitor, video camera, and headset. The patients captured their health data and answered 10 questions on nondialysis days according to an individualized care plan. Videoconferencing between the patient on hemodialysis and “a remote care nurse” was provided using the video camera and headset at arranged times. A trained remote nurse reviewed all incoming data and contacted the patients and caregivers via telephone if the data were outside the normal range or incomplete, as predetermined in the individual plan. The nurses provided additional technical support [62].

#### Vidyo

This is a secure videoconferencing platform used by behavioral therapists for sessions of live 2-way, technology-assisted, video-recorded cognitive behavioral therapy for patients on hemodialysis. Each session lasts 45 to 60 minutes. Patients use a study laptop with headphones and a microphone and connect in real time with therapists over a secure Wi-Fi hot spot. The platform is used for 8 regularly scheduled sessions per patient during regularly scheduled hemodialysis sessions [55].

#### Video or Telehealth Consultations in General

The telehealth platforms used by the participants in 4 nephrology clinics in the United States and Australia were not technically described [41,44,59]. In these studies, telehealth or telemedicine was determined following the World Health Organization’s definition: “...the provision of healthcare services at a distance... conducted between remote healthcare users seeking health services and healthcare providers (client-to-provider telemedicine)” [44,63]*.* Telehealth consultations occurred via secure 2-way technology-assisted platforms mediated through mobile phones, PCs, and tablets [21].

#### Association With EHRs

The association between the eHealth interventions and EHRs was inconsistently described in 2 patient portals: RPV and RelayHealth (Table 2). The descriptions stated that RPV allowed patients to gain secure access to parts of the EHR [53,54,58]. RPV is a written communication platform, and the study investigated the feedback system; however, it lacked a description of its association with the EHR [54,58]. The RPV portal provided access to blood test results from patient records [54]. Patients and HCPs could directly view the EHR. RelayHealth allowed both parties to see the messaging history and provided direct access and linkage to the EHR [57]. We were unable to extract data on the relationship between the telephone and videoconferencing platforms [41,44,55,59] or VitelCare [56] and EHRs.

#### Level of Patient Participation

The participative role of patients receiving hemodialysis was variously described in the 9 included studies (Table 2). In total, 44% (4/9) of the studies on the 2 patient portals described the possibility for written (email) asynchronous 2-way communication [53,54,57,58]. A total of 44% (4/9) of the studies on videoconferencing platforms described the possibility for oral, (partly) preagreed, and synchronous 2-way communication [41,44,55,59]. One study described a home telehealth monitoring unit with concomitant remote care nurse support—oral and synchronous 2-way communication at prearranged times [56]. VitelCare was vaguely described in the included articles. Therefore, we included additional information about VitelCare from the protocol article by Berman et al [62]—VitelCare also allowed for written, asynchronous communication (eg, email).

In all interventions, patients were involved to varying degrees, depending on the degree of patient involvement, based on our chosen taxonomy [30]. Patients were involved at level 1 (“information giving”) in 33% (3/9) of the studies [53,54,58]. This 33% (3/9) of the studies examined the RPV patient portal; however, they examined different populations at different periods and had different aims in examining the experience of patients with CKD regarding RPV use [53,54,58]. Therefore, they highlighted different relevant patient experiences. In total, 56% (5/9) of the studies [41,44,55,57,59] involved patients at level 2 (“consultation”), and 11% (1/9) of the studies [56] involved patients engaged in both asynchronous and synchronous communication, rated as level 2.

### Facilitators of and Barriers to Patient Participation in eHealth Communication Interventions

#### Overview

We were able to identify the facilitators of patient participation in all the included studies (Table 3) and the barriers to patient participation in 67% (6/9) of the studies [41,44,53,56,58,59] (Table 4).

**Table 3:** Facilitators to patient participation. The 4 categories are availability of and access to information; security, trust, and confidence; patient knowledge of their health situation; and preparedness for the future health situation and be able to talk about it with significant others

Study	4 overarching categories of facilitators			
	Availability of and access to information	Security, trust, and confidence	Patient knowledge of their health situation and use for self-care	Preparedness for the future health situation and be able to talk about it with significant others
Chu et al [59], 2023; part 1: from surveys	To have the necessary resources AND flexible telehealth options To have a stable internet connection	To have support for using telehealth To attend an appointment when it was arranged using telehealth To be confident using the technology	Feeling comfortable talking about health issues via telehealth	—^a^
Chu et al [59], 2023; part 2: from interviews	—	Reduces travel burden Telehealth consultations are convenient Provides flexibility	—	A belief that telehealth benefits the individual Telehealth reduces stress for HCPs^b^ and positively impacts the health care system Patients want to do what is expected of them
Giles et al [58], 2017; phase 1	Easy access to a computer Availability of free-text option	To feel confident in reporting Anonymity Feedback from reports Being able to give positive feedback	Leads to improvements	Encourage openness between staff and patients
Giles et al [58], 2017; phase 1 continued	—	Being confident that the feedback is going to the right person Belief that the feedback system will encourage improvement Independent voice for patients when giving feedback	—	—
Hazara and Bhandari [53], 2016	—	—	Check blood test results The availability of RPV^c^, particularly during travel, is a safety concern The availability despite infrequent use	Be prepared (have knowledge) to discuss their own situation with their physician
Hudson et al [54], 2020	—	—	Supporting ways of knowing Extending existing practice: to be in charge Translating information into practice	Engaging family, involving carers, and explaining health problems to carers Engaging HCPs and achieve an individual acceptance of ownership for health
Jakubowski et al [55], 2019	1-click access Technical logistical support from a person	Discretion (headphones, screens, and separate rooms)	“It gives me a chance to get some stuff off my back” Being able to talk one-on-one with someone was helpful	—
Kiberd et al [57], 2018	The portal was easy to use	—	—	Positive impact on access to kidney specialist
Ladin et al [41], 2021	More convenient, less costly, and more efficient	Reducing infection risk: telehealth consultations reduce face-to-face contact	—	Care partner engagement facilitates participation in consultations The clinician is more dedicated during a web-based session To be relaxed in one’s own home
Lew et al [44], 2023	Easy access to and availability of the videoconference because renal dietitians facilitated them at the bedside	Not concerned regarding internet security, privacy, or technical issues Spending enough time with their physician during web-based visits	Can address issues and obtain answers to medical questions	—
Minatodani and Berman [56], 2013	—	—	Being empowered and gaining a sense of control Self-awareness of health and learned illness self-management skills	A reminder of how the health situation impacts the caregiver

^a^Not applicable.

^b^HCP: health care professional.

^c^RPV: Renal Patient View.

**Table 4:** Barriers to patient participation. The 4 categories are unavailability of and challenging access to information, mistrust and unsafety, challenges in knowing their health situation and self-care, and relational challenges.

Study	4 overarching categories of barriers			
	Unavailability of and challenging access to information	Mistrust and unsafety	Patients’ challenges in knowing about their health situation and self-care	Relational challenges
Chu et al [59], 2023; part 1: from surveys	Lack of training or instructions on how to use telehealth Failing to understand how to use telehealth	—^a^	—	—
Chu et al [59], 2023; part 2: from interviews	Hearing Language and body language (eg, lip reading)	Concerns about the ability to perform routine assessments (eg, leg fluid) Mistrust in the clinician’s ability to do a physical examination	Entrenched negative attitudes and views regarding telehealth	Depersonalization HCPs^b^ lacking will or training negatively impact use
Giles et al [58], 2017	Language Computer literacy and access to a computer	Desire for an independent person to investigate the reports Concerns about repercussions for staff if the patient makes a negative report Perception that the information will not be dealt with or taken seriously	A negatively loaded name of the intervention Patients want to forget about dialysis when at home	Prefer face-to-face contact for feedback Fear of deteriorating relationships with HCPs Lack of support from relatives
Hazara and Bhandari [53], 2016	Design of website Log-in problems, loss of log-in credentials, and not knowing who to contact Access to a computer or the internet	Personal information may be seen by others	“Seeing my results online makes me more anxious” Being too busy to use the website Patients only use the website to check blood test results	A feeling that the intervention did not add anything to the patient’s existing relationship with the HCP
Hudson et al [54], 2020	—	—	—	—
Jakubowski et al [55], 2019	—	—	—	—
Kiberd et al [57], 2018	—	—	—	—
Ladin et al [41], 2021	Lack of a private, quiet place Technical and medical literacy challenges Not knowing who to contact in the event of technical problems	A clinician’s diagnosis is challenging to trust when both parties do not meet face-to-face	Measuring blood pressure at home is difficult Hearing loss and loss of face mimics make web-based communication challenging	Loss of interpersonal connection and feeling alone
Lew et al [44], 2023	—	Concerns that the lack of a physical examination hampered the physician’s ability to care for them	—	—
Minatodani and Berman [56], 2013	Technical challenges (eg, outdated software)	—	Feeling too sick, weak, or tired Forgetting to send measurements	—

^a^Not applicable.

^b^HCP: health care professional.

#### Facilitators

The first facilitator (Table 3) was related to availability of and access to the intervention and included easy access to computers and 1-click access, support for log-in problems, and less costly and more efficient use. The second facilitator was related to security and confidence and included being confident that the feedback went to the right person, feeling confident about reporting, discretion (eg, a separate room), and reducing infection risk. The third facilitator addressed patient knowledge of their health situation, including checking blood test results, self-awareness of health, and the availability of the patient portal during travel. This facilitator also included self-care skills, such as translating information into practice, one-to-one talks, and gaining a sense of control. The fourth facilitator addressed preparedness related to changes in health situations and skills in relation to others, family and friends, and HCPs.

#### Barriers

The first barrier (Table 4) addressed the challenges regarding the availability of and access to the intervention and included physical challenges such as hearing, understanding the language and body language, lack of access to computers or the internet, loss of log-in credentials, and lack of a private place. The second barrier was related to mistrust and unsafety and included worries that personal information may be seen by others, concerns about staff repercussions if the patient made a negative report, the perception that the information would not be dealt with, and worries that a clinician’s diagnosis would be challenging to trust when patients and physicians do not physically meet. The third barrier concerned knowledge of the health situation and self-care and included a desire to forget about dialysis at home; anxiety about seeing the measured blood pressure on the web would lead; difficulty measuring blood pressure at home; and feeling too sick, weak, or tired. The fourth barrier addressed the relational challenges for people undergoing hemodialysis concerning other people (eg, HCPs and family members). In addition, it addressed that HCPs lacking will or training in facilitating telehealth consultations negatively impacted their use for the patients. The results indicated preferences for face-to-face contact for feedback, lack of support from relatives, a feeling that the intervention did not add anything to the patient’s existing relationship with the HCP, and hearing loss and loss of face mimics causing challenges in web-based communication.

## Discussion

### Principal Findings

This scoping review aimed to identify and map the available evidence on patient participation in eHealth communication interventions. We identified 4 specific patient portals or conferencing platforms and 3 nonspecified digital audio or video solutions used in eHealth consultations, all used and evaluated through various study designs involving patients undergoing hemodialysis. We found that eHealth communication intervention types were inadequately described, including their link to the EHR. The level of patient participation was predominantly low. Key barriers to participation included unavailability of and limited access to information, mistrust and feelings of insecurity, lack of knowledge about their health situation and self-care, and relational challenges. Conversely, key facilitators of participation were availability of and access to information; security, trust, and confidence; patients’ knowledge of their health situation and use for self-care; and preparedness for future health events and explain situation to significant others.

### Few Possibilities for Patient-Oriented or Customized Digital Communication and Lack of 2-Way Visibility of Information in EHRs

We only identified 4 specified eHealth communication interventions; nevertheless, the small number of interventions provided different opportunities, both asynchronous and synchronous, for 2-way communication. The lack of description of the interventions’ concrete opportunities for 2-way communication obscured the participative role of patients undergoing hemodialysis in digital communication. Only Minatodani and Berman [56] described how the monitoring unit offered both asynchronous and synchronous communication; however, they only vaguely described the functionality without a link to the EHR. No further descriptions were found for the 7 included interventions, and the combined possibility for asynchronous and synchronous communication contributed minimally to modeling future 2-way eHealth communication interventions. Therefore, further research is required in this area that aligns well with the general literature on the subject as a complex eHealth landscape [7].

The patients’ low participative role, as evidenced by their hesitation to use or be consistent users of eHealth interventions, may be attributed to a perception of digital interventions as complex, as indicated by our barrier analysis. Regarding barriers, we found that both technical challenges and mistrust may contribute to preventing patients undergoing hemodialysis from using eHealth interventions. This is in line with the experience of patients with CKD who initially had access to but did not persist in using their patient portal [28]. Communication must be patient centered to be therapeutic [64]. Communication can result in a more collaborative relationship between patients and HCPs in which the patients’ voice is heard and respected. Our results show that patients undergoing hemodialysis desire a therapeutic and collaborative relationship with their HCPs, and we found no reason to assume that the same desire does not apply to digital communication. Patients may feel disempowered and unheard if patient-centeredness is not adopted in digital communication with HCPs. This can lead to a breakdown in trust and a less collaborative patient-provider relationship.

To overcome the barriers to patient participation at a level higher than that identified in this review [30], we propose that patients undergoing hemodialysis need to feel safer using eHealth communication interventions. Communication misunderstandings have been documented as a major safety risk for patient harm in renal units [65], and patients require reassurance, education, and ongoing support to view eHealth interventions as an aid to improve communication. Developing a culture of patient safety demands multifaceted efforts; nevertheless, the responsibility to systematically ensure patient safety falls on HCPs and the health organization [66,67]. In addition, patients must find it more meaningful to use eHealth communication interventions and should view the interventions as a modality in addition to or aligned with their existing physical relationship with their HCP. A relational barrier to patient participation in eHealth communication was “...a feeling that the intervention did not add anything to the member’s existing relationship with HCP” [53]. Thus, the few interventions that offered asynchronous digital communication, such as email correspondence, offered patients minimal opportunities for higher levels of participation [30]. An example of dissatisfaction with 1-way communication is the desire of patients undergoing hemodialysis for 2-way communication and feedback on their ePROMs, highlighting their desire for communication and relationships with HCPs [25,68]. This desire for 2-way communication has also been documented in general patient populations [69].

One important finding is the lack of detailed descriptions of what patients can view in their EHRs and their experiences with this access. In addition, we were unable to draw any conclusions on how patients perceive the information provided in the EHR and their potential requirements for future interventions. This knowledge gap may also include the challenges that patients face in understanding the medical content in the EHR. We found examples of patients not being able to understand the information in the EHR. The barrier “seeing my result online makes me more anxious” [53] may indicate that the patients may require having their results explained by the HCP and discussing their results with them. The benefits of combining the asynchronous and synchronous functionalities of eHealth communication interventions remain undercommunicated, and the aims and functions of physical and digital communication remain underdiscussed.

### Low Levels of Patient Participation

The low levels of patient participation (Table 2) can indicate a passive patient role and limited opportunities to be active, ask questions, demand, or make decisions [30]. However, a low level of patient participation can also indicate that patients do not make decisions about their own situation despite the opportunity for more engagement. The barriers and facilitators in this study do not indicate an explicit formula for eHealth communication interventions to increase engagement by all patients. Higher levels of patient involvement (Table 2), as described by Thompson [30], imply 2-way communication. The highest levels include dialogue, shared decisions, and transmission of power and knowledge from the HCP to the patient [30,31]. Our results show that eHealth use may lead to patients’ fear of increased distance from HCPs [41]. The low level of patient participation may be related to the power dynamic between patients and professionals [30,31].

Patients’ fear of increased distance from HCPs can further lead to a negative power balance, consistent with the results of a scoping review of good practices for dialysis education, treatment, and eHealth [70]. Further conceptualization and development of interventions may continue without real patient participation if patient experience is not accounted for in the development of evaluation instruments. The patients’ wish to maintain interactions and relationships with HCPs is in line with the importance of the nurse-patient interaction described by Berman et al [62]. We can assume that feeling safe and secure is a prerequisite for transition from physical to digital nurse-patient interaction. As described previously, the level of patient involvement and the balance of power between patients and HCPs may be negatively affected if eHealth interventions do not achieve their intended purpose: increased autonomy, a stronger patient voice, and more patient power [7]. Knowledge is an undeniable prerequisite for power [71].

### Barriers and Facilitators Related to Health Literacy

The EHR is one of the central sources of patient knowledge about their situation, and the lack of access to EHRs limits patient knowledge about their medical situation. Knowledge of one’s health is connected to the concept of health literacy, defined as *“*...the ability of an individual to obtain and translate knowledge and information in order to maintain and improve health in a way that is appropriate to the individual and system contexts” [72]. This definition includes three broad themes that capture the essence of the health literacy concept: (1) knowledge of health, health care, and health systems; (2) processing and using information in various formats in relation to health and health care; and (3) the ability to maintain health through self-management and working in partnership with health providers [72]. We aimed to demonstrate how our findings on barriers and facilitators may provide insights into the multifaceted approaches required to increase patient participation and health literacy in the hemodialysis context [73]. The themes of the barriers and facilitators relate to different competencies in our understanding of the health literacy concept [74].

First, our findings on the barriers to and facilitators of “availability and access to information” and “trusting the security of the interventions” relate to the first concept of health literacy, that is, “to be able to receive knowledge*.*” The results of the RPV patient portal are in line with those of a study of nonusers of the same portal demonstrating that practical barriers (eg, access to the intervention and lack of assistance with the first log-in) are crucial barriers to persistent use [28]. Facilitators of the sustained use of patient portals include initial support and establishment of access to web-based records. The interventions must be aimed to facilitate a user interface arranged for and personalized to the individual user, with content generalized to the population [28]. Patients undergoing hemodialysis may have limited access to technology, such as smartphones or computers, causing difficulty in implementing digital communication tools. Considering that older adults account for most of the population undergoing hemodialysis worldwide [75] and older persons generally have greater challenges using eHealth, it is reasonable to assume that patients undergoing hemodialysis may require more support than other patient groups [76]. In addition, patients undergoing hemodialysis may have physical (eg, poorer vision and intention tremors) and cognitive (eg, mood and memory problems) limitations, causing difficulty in using certain types of technology [24,77].

HCPs and patients undergoing hemodialysis meet frequently, and HCPs are in a unique position to individualize support and motivation and appear safe and confident to increase both the technical and safe use of eHealth communication interventions among patients. However, HCPs must be awarded credit by their employers for their resources and time to perform this important part of their daily workflow, thereby facilitating alternative approaches to physical meetings. Access to EHRs by both patients and HCPs could contribute to shared knowledge and improved relationships. The low level of participation among patients undergoing hemodialysis in this study correlates with the self-reported level of health literacy among the population in a Norwegian study, in which older persons and persons with chronic diseases had a lower level of health literacy [78]. We can assume that this result is comparable with those in other countries. Therefore, efforts to provide the population undergoing hemodialysis with digital possibilities to acquire knowledge about their own health situation may demand more resources than those for other populations (eg, younger populations).

Second, we found that the barriers to and facilitators of “security and trust” are related partly to the first concept of health literacy and partly to the second concept, that is, “processing and using the information*.*” Discretion is an explicit barrier to patient participation. Discretion is a challenge when discussing sensitive information in an hemodialysis unit where several patients concurrently undergo hemodialysis. The possibility of digitally exchanging information and knowledge that can be read and processed in private, combined with digital or physical consultations, may be a fruitful solution. A special challenge when promoting digital communication for patients undergoing hemodialysis is their close relationship with their HCPs, and digital communication must offer value that is not obtained through physical meetings. Both patients and HCPs must find worthy interventions. Moreover, the sharing of data about health situations, treatment, results, and challenges can facilitate a higher level of knowledge, which is related to a higher level of patient participation [30] and health literacy [72]. In addition, a higher level of knowledge can facilitate shared understanding through further face-to-face or digital discussions.

Third, we found that the barriers to and facilitators of “preparedness and relation to others” related to the third concept of health literacy: “ability to maintain health through self-management and working in partnerships with health providers.” An explicit barrier was that the intervention did not add anything positive to the existing relationship with the HCP [53,58]. The relationships between patients undergoing hemodialysis and HCPs are long term, and patients have a dependent relationship with the HCP. This correlates with a review describing that older people with multimorbidity regard a lack of self-efficacy, knowledge, support, functionality, and information provision regarding the benefits of eHealth as the main barriers to using eHealth [29]. Our review confirmed that important facilitators for improving patient participation include active engagement of target end users in the design and delivery of eHealth programs, support for overcoming privacy concerns, and enhancement of self-efficacy in the use of technology. The integration of eHealth applications and programs across health services to accommodate the patient group of older adults with multimorbidity may be a key facilitator [29].

Patient involvement was challenged for various reasons, including technical issues, safety, self-care, and relational factors, as identified and presented in the overarching categories of barriers and facilitators (Tables 3 and 4). This means that challenges that hinder increased patient involvement require action at different levels. Similarly, a multifaceted approach is required to address the challenges within all 3 broad themes and levels of health literacy. This is in line with the understanding that health literacy encompasses different levels and skills and can be developed through education, particularly if it is well conceptualized and context specific [79]. We support an encompassing approach that includes overcoming barriers and strengthening facilitators to support greater autonomy and personal empowerment in the hemodialysis context.

### Limitations

This scoping review solely identified and mapped the current evidence regarding patient participation in eHealth communication interventions. The findings do not offer recommendations for practice or research but rather highlight research gaps and inspire future studies. According to the Joanna Briggs Institute scoping review guidelines [33], assessing the methodological quality of the included studies was not applicable. However, this review’s primary strength is its comprehensive systematic searches across multiple databases. Despite identifying only 9 studies on eHealth interventions, we are confident that these studies represent the relevant published sources on patient participation in eHealth communication interventions. Adherence to the standardized a priori protocol [31] and the PRISMA-ScR reporting guidelines [46] aided in ensuring consistency and transparency in the review. One limitation may be that the studies’ descriptions of patient involvement were sparse, and we may have misjudged the degree of patient participation. To counteract this, we relied on supplementary sources for more detailed descriptions of the interventions. The predominance of qualitative designs may preclude obtaining valid and honest answers, particularly during oral evaluations. This is particularly true for the frail population of older adults undergoing hemodialysis, who may feel dependent on their relationships with HCPs [55]. All the studies reported facilitators (9/9, 100%) but did not mention barriers (3/9, 33%). However, participants in qualitative research projects may find it challenging to provide objective and explicitly negative evaluations orally or face-to-face.

### Implications for Future Research and Practice

This review highlighted the current understanding and gaps in knowledge regarding patient participation in eHealth communication interventions for patients undergoing hemodialysis. We emphasize the need for diverse research methodologies, including qualitative, quantitative, and mixed methods, to explore patient experiences and evaluate the use of these interventions. Research should inform the design, innovation, and implementation of eHealth projects, ensuring compliance with ethical considerations, to uncover both positive and negative feedback. The needs of patients may vary across different stages of CKD and types of renal replacement therapy, presenting challenges when diverse needs are assessed within the same group. Stakeholder perspectives, including patients, HCPs, and caregivers, should be integrated into cocreational processes to develop accessible and safe eHealth communication models. Research-based interventions should systematically address barriers and enhance patient participation across various health literacy themes. In addition, the health care system must facilitate eHealth communication as part of HCPs’ daily workflow supplemented by face-to-face interactions. Finally, additional research is required to fulfill the aim of increased HHD.

### Conclusions

Patients undergoing hemodialysis require 2-way communication and relationships with HCPs. Low patient participation is associated with low levels of health literacy in other older populations with chronic illnesses. Working at different levels to overcome barriers to patient participation can facilitate higher levels of patient participation and health literacy. HCPs should be the preferred facilitators of eHealth communication with patients undergoing hemodialysis to improve feelings of safety and security. Facilitating shared knowledge, patient participation, and health literacy is vital to improving the visibility of EHR data for patients.

## Appendix

Multimedia Appendix 1 PRISMA (Preferred Reporting Items for Systematic Reviews and Meta-Analyses) checklist.

## Abbreviations

CKD: chronic kidney disease
EHR: electronic health record
ePROM: electronic patient-reported outcome measure
HCP: health care professional
HHD: home hemodialysis
PD: peritoneal dialysis
PRISMA-ScR: Preferred Reporting Items for Systematic Reviews and Meta-Analyses extension for Scoping Reviews
QOL: quality of life
RPV: Renal Patient View
RQ: research question

## Data Availability

All data generated or analyzed during this study are included in this published article and its supplementary information files.
